# The Rescue of miR-148a Expression in Pancreatic Cancer: An Inappropriate Therapeutic Tool

**DOI:** 10.1371/journal.pone.0055513

**Published:** 2013-01-31

**Authors:** Yannick Delpu, Hubert Lulka, Flavie Sicard, Nathalie Saint-Laurent, Frédéric Lopez, Naïma Hanoun, Louis Buscail, Pierre Cordelier, Jérôme Torrisani

**Affiliations:** 1 INSERM UMR 1037- University of Toulouse III, Cancer Research Center of Toulouse (CRCT), University Hospital Center Rangeuil, Toulouse, France; 2 Paul Sabatier University, Toulouse, France; 3 Department of Gastroenterology, University Hospital Center Rangueil-Larrey, Toulouse, France; 4 Basic and Clinical Proteomics Group, INSERM UMR 1037, Cancer Research Center of Toulouse, University Hospital Center Rangueil, Toulouse, France; Technische Universität München, Germany

## Abstract

MicroRNAs are small non-coding RNAs that physiologically modulate proteins expression, and regulate numerous cellular mechanisms. Alteration of microRNA expression has been described in cancer and is associated to tumor initiation and progression. The microRNA 148a (miR-148a) is frequently down-regulated in cancer. We previously demonstrated that its down-regulation by DNA hypermethylation is an early event in pancreatic ductal adenocarcinoma (PDAC) carcinogenesis, suggesting a tumor suppressive function. Here, we investigate the potential role of miR-148a over-expression in PDAC as a therapeutic tool. We first report the consequences of miR-148a over-expression in PDAC cell lines. We demonstrate that miR-148a over-expression has no dramatic effect on cell proliferation and cell chemo-sensitivity in four well described PDAC cell lines. We also investigate the modulation of protein expression by a global proteomic approach (2D-DIGE). We show that despite its massive over-expression, miR-148a weakly modulates protein expression, thus preventing the identification of protein targets in PDAC cell lines. More importantly, *in vivo* data demonstrate that modulating miR-148a expression either in the epithelia tumor cells and/or in the tumor microenvironment does not impede tumor growth. Taken together, we demonstrate herein that miR-148a does not impact PDAC proliferation both *in vitro* and *in vivo* thus suggesting a weak potential as a therapeutic tool.

## Introduction

Pancreatic ductal adenocarcinoma (PDAC) is the fourth leading cause of death by cancer in Western countries whereas it represents only 3% of new cases each year [1]. PDAC prognosis is frequently explained by a lack of early specific diagnostic markers and by the absence of efficient treatments. To date, surgery represents the only curative approach for PDAC management but concerns a small subset of patients due to PDAC aggressiveness and invasive phenotype. For the remaining patients diagnosed with locally advanced or metastatic PDAC, gemcitabine chemotherapy is the standard palliative treatment with modest effect on survival [2]. Consequently, remarkable studies have been performed to elucidate the key events driving pancreatic carcinogenesis, to identify new targets and to develop tumor-targeted therapies [3], [4]. Genetic and epigenetic alterations have been described as an early event in the development of PDAC [5]. In the last decade, significant works highlighted the impact of such molecular alterations on microRNAs expression in PDAC. MicroRNAs are small non coding RNAs that inhibit the translation of their target mRNAs. They play important roles in cancer development, invasiveness and resistance to chemotherapies [6].

We previously demonstrated that microRNA-148a expression (miR-148a) is down-regulated early during PDAC development by hypermethylation of its genomic DNA sequence [7]. Other studies reported the down-regulation of miR-148a expression in other cancers (*i.e.*: colorectal, esophageal, gastric cancers and cancer metastasis) [8], [9], [10], [11]. Several miR-148a mRNA targets were described in different types of cancer. These targets regroup cell cycle, apoptosis or DNA methylation effectors.

Previous studies evoked a tumor suppressive effect consequent to an over-expression of miR-148a in colorectal and gastric cancer-derived cell lines [8], [10]. Consequently, the present study aimed to determine whether restoring miR-148a expression impacts PDAC proliferation both *in vitro* and *in vivo* and therefore could be proposed as a therapeutic tool.

## Materials and Methods

### Cell culture

Human PDAC Capan-2 and IMIM-PC2 cell lines was grown in RPMI medium supplemented with 100 mL/L fetal calf serum, L-glutamine (2 mM) (Life Technologies), antibiotic and antimycotic cocktail (Life Technologies) and Plasmocin® (5 µg/mL) (InvivoGen). MIA PaCa-2 and PANC-1 PDAC cells and Human Embryonic Kidney HEK-293FT cells were grown in DMEM containing 4.5 g/L glucose (Life Technologies), 100 mL/L fetal calf serum, L-glutamine, antibiotics, Fungizone® and Plasmocin® (InvivoGen). Human PDAC-derived BxPC-3 cells were grown in DMEM containing 1 g/L glucose (Life Technologies), 100 mL/L fetal calf serum, L-glutamine, antibiotic and antimycotic cocktails. Human Pancreatic Nestin positive Epithelial cells (HPNE) were grown in 75% DMEM 4.5 g/L glucose (Life Technologies), 25% Medium M3 Base (Incell Corp.), fetal bovine serum 5%, 10 ng/mL human recombinant EGF (Sigma-Aldrich) and 750 ng/mL gentamycin (Life Technologies). All cell lines were obtained from the American Type Culture Collection (ATCC) excepted for HPDE cells and HPNE cells which are kind gifts from Dr M.S. Tsao (University of Toronto, Canada) and Dr M. Ouellette (University of Nebraska Medical Center, Omaha, USA), respectively [12] [13]. IMIM-PC2 cells were obtained from Dr FX Real (Spanish National Cancer Research Centre, Madrid, Spain).

### Cell transfection

For all *in vitro* experiments, miR-148a pre-miR™ miRNA precursors (Ambion) at 25 nM and 50 nM were transfected using siPORT™ NeoFX™ Transfection Agent (Ambion) according to manufacturer's instructions. Cy™3 dye-labeled Pre-miR was used as negative control and was transfected using the same conditions.

### Cell cycle analysis by flow cytometry

Transfected Capan-2 cells were collected, rinsed once in phosphate-buffered saline (PBS) and fixed in ice-cold 70% ethanol overnight at 4°C. Cells were collected by centrifugation at 1.000 g and rinsed with PBS. Cells were labeled with propidium iodide (Life Technologies) following manufacturer's recommendations. Cell cycle distribution was assessed using BD FACS Calibur apparatus (Beckton Dickinson) and Cell quest pro software (Beckton Dickinson) as described by Hanoun *et al.* [14].

### 2D-DIGE electrophoresis

Cell lysis was performed in Urea 8M, Thiourea 2M, CHAPS 4%, pH 8.5. After clean-up precipitation (2D Clean Up kit, GE Healthcare), proteins were resuspended in 2D-DIGE sample buffer (Urea 8M, Thiourea 2M, CHAPS 4%, pH 8.5). Protein concentration was determined with 2D Quant kit (GE-Healthcare). Fifty micrograms of proteins were labeled with 400 pmol of CyDye DIGE Fluor Minimal Dyes (GE Healthcare) and incubated on ice in the dark for 30 min according to manufacturer's instructions. The reaction was stopped by the addition of 1 µl of 10 mM lysine and incubated on ice for 10 min. The differentially Cy-3 and Cy-5 labeled samples were mixed with the Cy-2 labeled internal standard (sample composed of equal aliquots of each sample of the experiment) and IsoElectric Focusing (IEF) re-hydratation buffer (Urea 8M, Thiourea 2M, CHAPS 2%, 10 mM DTT, 1,2% IPG (Immobilized pH Gradient) buffer, pH 4–7, GE Healthcare) was added up to 350 µL. For the 2D-DIGE analysis, IEF was performed using an IPGPhor 2 apparatus (GE Healthcare) according to the manufacturer's recommendations with immobilized pH gradient (IPG) pH 4–7, 18 cm. Strips were first incubated in equilibration buffer (Urea 6 M, SDS 2%, Tris-HCl 50 mM pH 8.6, glycerol 30%, bromophenol blue trace) containing 1% DTT for 15 min and thereafter in the same buffer with 4.5% iodoacetamide, for 15 min, in the dark. The strips were loaded on the top of 12.5% acrylamide gels and ran at 1 W/h for 1.5 h and at 15 W/h until the bromophenol blue reaches the bottom-end of the gel. The gels were scanned using a Typhoon trio Imager (GE Healthcare) at 100 µm resolution with λex/em of 488/520, 532/580, and 633/670 nm for Cy-2, Cy-3 and Cy-5, respectively. Image analysis was performed using DeCyder 6.5 software (GE Healthcare).

### Plasmids

The pLVMND-SLuc plasmid encoding the secreted Gaussia Luciferase (Gluc) was kindly provided by Cayla-InVivoGen (Toulouse, France). Lentiviral expression vector encoding miR-148a and the copGFP (pMIRNA1-miR148a) or copGFP alone (pMIRNA1-GFP) were obtained from Biovalley (Marne-la-Vallée, France). Plenti6-TR, encoding the TET repressor, was obtained from Life Technologies. For the inducible expression of miR-148, two partially complementary oligonucleotides corresponding to mature miR-148a or a control scrambled miR (miR-CT) were hybridized and cloned into pcDNA6.2-GW/emGFP-miR vector (Life Technologies) before recombination into pLenti4/TO/V5 DEST vector (Life Technologies) using Gateway® strategy. For lentivectors production, packaging plasmids pHCMV-G, encoding for VSV-G protein, and pCMVΔ8.91, encoding for HIV-1 accessory proteins, were kindly donated by Dr A. Dubart-Kupperschmitt (Paris, France).

### Lentiviral vector production

All replication defective, self-inactivating lentiviral vectors were generated in a BSL-3 facility (Vectorology platform, INSERM U1037 Cancer Research Center of Toulouse, Toulouse, France) as previously described by Torrisani *et al.* [15]. Briefly, transient transfection of HEK-293FT cells with packaging and lentiviral vector plasmids were performed using calcium phosphate precipitation. pLenti4/TO/GFP-miR-148a, pLenti4/TO/GFP miR-CT were used to obtain lentiviral particles encoding inducible miR-148a or miR-CT (namely, LV-TO-miR-148a or LV-TO-miR-CT, respectively). On the other hand, pMIRNA1-miR148a and pMIRNA1-GFP were used to obtain lentiviral particles constitutively encoding miR-148a or miR-CT (LV-miR-148a or LV-GFP, respectively). Plenti6-TR and pLVMND-SLuc plasmids were used to obtain lentiviral particles encoding the Tet repressor or the secreted luciferase (LV-TR or LV-Gluc, respectively). All batches were verified replicative virus-free. The viral titers were determined on HT-1080 cells and expressed in transduction unit/ml (TU/ml) as described elsewhere. In addition, vector concentrations were quantified by p24 ELISA (Innotest, Ingen, Paris).

### Generation of MIA PaCa-2 stable cell line over-expressing miR-148a

MIA PaCa-2 cells were incubated with the LV-TR lentiviral particles (multiplicity of infection = 5) for 24 h and subsequently selected with blasticidin (Invivogen, Toulouse, France) at 100 µg/mL for 2 weeks and cloned by serial dilution to obtain MIA PaCa-2 TR cells. MIA PaCa-2 TR cells were further transducted by LV–Gluc (multiplicity of infection = 5) and cloned by serial dilution to give MIA PaC-2 TR–Gluc cells. The latter cells were incubated with LV-TO-miR-148a or LV-TO-miR-CT (multiplicity of infection = 5) for 24 h and selected with zeocin (Invivogen) at 100 µg/mL for 3 weeks and cloned by serial dilution to generate MIA PaCa-2 TR–Gluc miR-148a and MIA PaCa-2 TR–Gluc miR-CT cell lines, respectively. An optimal doxycyclin concentration of 1 µg/mL was determined to induce a maximal expression of miR-148a. The different stable cell lines were then constantly grown in the presence or the absence of doxycyclin.

### Measurement of miR-148a expression by qRT-PCR

Cells were first transiently transfected or stably transducted as described above. Total RNA was isolated from cell lines with TRIzol Reagent (Life Technologies) according to supplier's instructions. RNA concentration was measured with a NanoDrop ND-1000 spectrophotometer (Thermo Scientific). The miScript PCR System (Qiagen) was used according to the manufacturer's instructions to quantify mature microRNAs from 1 µg of total RNA. U6 and 5S RNAs were used as internal controls. cDNA samples were diluted 1 into 100 for microRNA detection or U6 and 1 into 10.000 for 5S RNA detection. Duplicate qRT-PCR assays were carried out in a StepOnePlus™ Real-Time PCR System (Applied Biosystems) with SYBR Green PCR Master Mix (Qiagen). Relative amounts of miRNA were calculated by the comparative threshold cycle (CT) method as 2–ΔCT, where ΔCT = CTmiRNA – CT geometric mean of U6 and 5S.

### Proliferation assay

MiR-148a precursor or Cy™3 dye-labeled Negative Control precursor transfected cells were seeded at 6×10^3^ cells per well of a 96 well dish and grown in complete medium. Number of viable cells was determined by colorimetric method using CellTiter 96® AQueous Non-Radioactive Cell Proliferation Assay (Promega) according to manufacturer's instructions at day 4. For proliferation measurement, cells were seeded at 5×10^4^ cells per well of a 35 mm dishes. Cells were grown in complete medium supplemented with 1 µg/mL of doxycycline. Five hundred nanograms of doxycycline were added at 48 h to prevent its decay in culture medium. Cells were counted with a Coulter counter model ZM (Beckman Coulter) after 24 h, 48 h, 72 h and 96 h of culture.

### Gemcitabine sensitivity assay

Twenty five thousand exponentially growing MIA PaCa-2 cells stably expressing miR-148a or control microRNA grown with or without doxycycline were plated in 35 mm dishes. Twenty four hours later, cells were treated with gemcitabine (Eli Lilly) at different doses ranging from 1×10^−9^ M to 1×10^−4^ M. After 72 h of treatment, cells were counted using Coulter counter model ZM (Beckman Coulter). Gemcitabine “Lethal Concentration 50” (LC_50_) is the concentration of gemcitabine for which 50% of treated cells died comparatively to untreated cells at 96 hours. For the measurement of gemcitabine sensitivity in cell transiently over-expressing miR-148a, 1000 of exponentially growing PDAC cells transiently over-expressing miR-148a or a control microRNA (miR-CT) were plated in 96 well plate. Cells were treated with different doses of gemcitabine ranging from 1×10^−9^ M to 1×10^−4^ M for 72 h. For each cell line, cell viability was assessed by colorimetric method, compared to viability measured in 1×10^−9^ M treated wells and represented as a percentage of surviving cells.

### Migration and invasion assays

One hundred thousand exponentially growing cells over-expressing miR-148a or the GFP reporter protein were serum-starved for 24 h and seeded into 24 well-plate sized inserts (8 µm porous 24 well format inserts for migration assays and Biocat Matrigel invasion chambers for invasion assays, BD Falcon) according to manufacturer instructions. After 15 h, migrated cells were stained with a 1% crystal violet in 20% methanol solution, lysed and cellular density was determined by optical density measure of cell lysates at 560 nm. Results are expressed as percentage of migrating or invading miR-148a over-expressing cells compared to GFP expressing cells.

### Orthotopic cell graft

Eight-week-old SCID/beige CB 17 leaky mice (TAKONIC) were used for cell graft experiments. Each mouse received 12×10^6^ of exponentially growing MIA PaCa-2 cells stably overexpressing miR-148a in 100 µL of PBS injected in the tail of the pancreas. Injections were performed with a 29 gauge lymphography catheter set with a 29 gauge insulin needle. Mice grafted with miR-148a expressing cells received water *ad libitum* supplemented with sucrose (25 g/L) and doxycycline (2 g/L). Control mice received water *ad libitum* supplemented with sucrose only (25 g/L). Thirty days after xenograft, mice were sacrificed and tumors were removed, weighed and measured. Tumor volume was determined by the formula ((Tumor Length)×(Tumor Width^2^))/2. This study was performed in strict accordance with the recommendations in the chart for the Care and Use of Laboratory Animals of the INSERM. All surgery was performed under isoflurane anesthesia, and every effort was made to minimize suffering. All protocols including animals were approved by the ANEXPLO ethic committee. Macroscopic observation of tumors was performed by a skilled pathologist.

### *In vivo* tumor transduction of MIA PaCa-2 tumors

Orthotopic tumors were established in SCID CB17 mice pancreas by injection of 12×10^6^ of exponentially growing MIA PaCa-2-Gluc (as described above). Tumors were grown for 15 days before injection of 250 ng p24 of LV-miR148a lentivector using a 29 gauge lymphography catheter set with a 29 gauge insulin needle. LV-GFP was injected in control mice. Tumor progression before and after transduction was monitored by measurment of Gluc level in mice serum (as described below) and by abdominal palpation.

### Luciferase activity in mice serum

Luciferase activity was measured from mice serum. Blood was collected from retro-orbital sampling using Pasteur capillary pipette (VWR) prefilled with 10 µL of a 20% EDTA solution. Blood samples were kept on ice until centrifugation. Samples were spun 3.000 g for 15 min at 4°C and serum were collected and stored −20°C until use. Luciferase activity was determined from 5 µL of serum and 45 µL of a 50 µM coelenterazine solution (Fluka analytical) on a Luminoskan ascent device (Thermo Scientific).

### Statistical analysis

Results are expressed as the mean ± Standard Error. The statistical significance of differences was measured with the Mann-Whitney non parametric test, with a *p* value<0.05 considered statistically significant. The symbol * indicates a *p* value<0.05, ** indicates a *p* value<0.01 and *** indicates a *p* value<0.005.

## Results

### Effect of transient miR-148a over-expression on PDAC cell line growth

**Figure 1:**
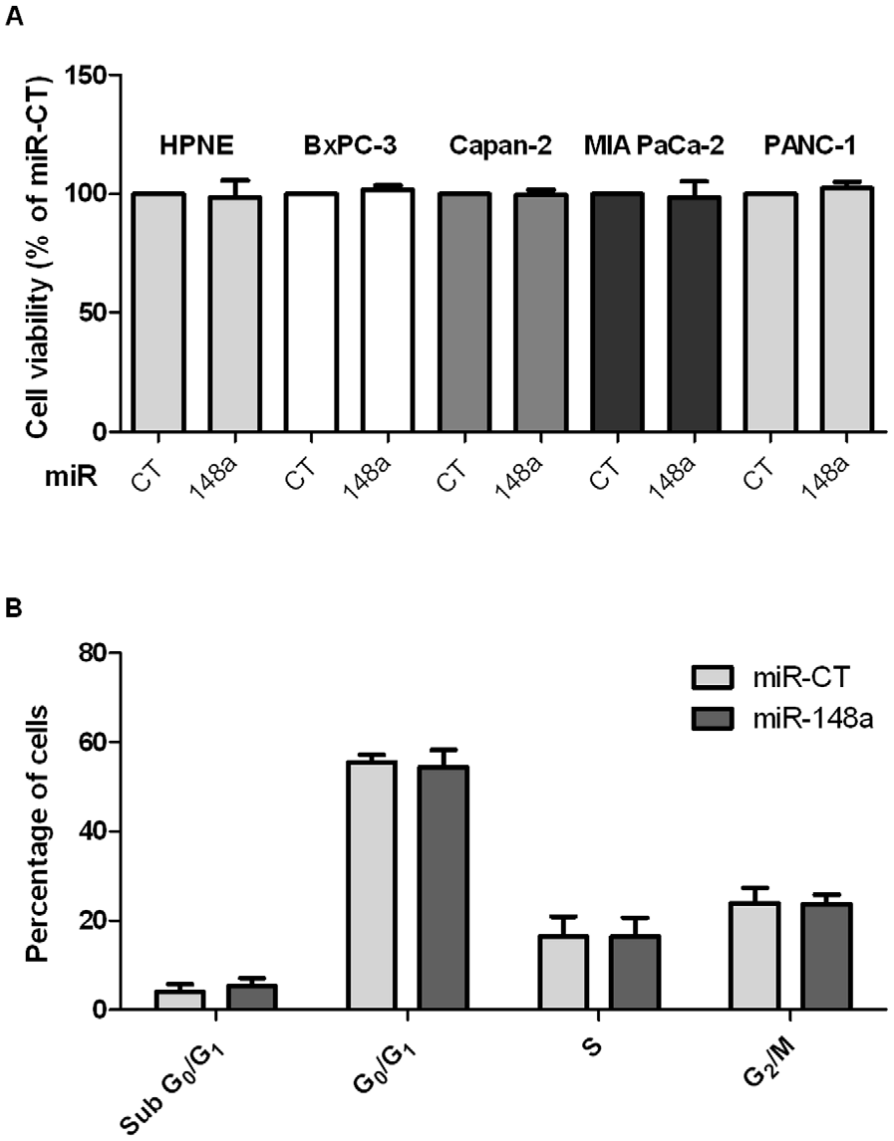
Transient over-expression of miR-148a in PDAC cell lines. (**A**) Capan-2, PANC-1, MIA PaCa-2 and BxPC-3 PDAC-derived cell lines and non-cancerous HPNE cell line were transiently transfected with miR-CT or miR-148a precursors. Four days after transfection, cell viability was assessed by colorimetric methods. For each cell line, the miR-148a-cell viability was compared to miR-CT cell viability (100%). The results are the mean of three independent experiments (±SEM) and are expressed as percentage of cell viability of control cells. (**B**), Cell cycle distribution was measured by propidium iodide staining followed by FACS analysis 72 hours after transfection of Capan-2 cells with miR-CT or miR-148a precursors.

We previously reported that miR-148a expression is repressed by DNA hypermethylation of its genomic sequence in PDAC cell lines and tumors [7]. One could speculate that the loss of miR-148a expression is crucial for PDAC development. To assess tumor suppressive effects of miR-148a in PDAC, Capan-2, PANC-1, MIA PaCa-2 and BxPC-3 PDAC cell lines were transfected by a miR-148a oligonucleotide precursor (miR-148a) or a control precursor (miR-CT). HPNE cells which are human pancreatic cells displaying a normal phenotype were used as “non-cancerous” control cells. Cell proliferation was measured by colorimetric method 4 days after transfection. MiR-CT transfected cells were used as internal reference for each cell line (Figure 1A). Transfection efficiency was assessed for each cell line by Cy3 fluorescence measurement and reached 90–100% (data not shown). Resulting miR-148a expression was measured by qRT-PCR (Figure S1A). MiR-148a over-expression in HPNE pancreatic normal cells did not induce significant changes in cell proliferation. Similarly, no significant change in cell proliferation was observed in the four PDAC cell lines compared to Cy3 control transfected cells. In parallel, cell cycle distribution in Capan-2 cells was determined by FACS analysis (Figure 1B). According to the absence of effect concerning cellular proliferation, no alteration in cell cycle distribution was observed in miR-148a transfected cells compared to miR-CT cells. These results indicate that transient miR-148a over-expression has no particular effect on cell proliferation of four PDAC cell lines.

### Effect of stable miR-148a over-expression on PDAC cell line behavior

**Figure 2:**
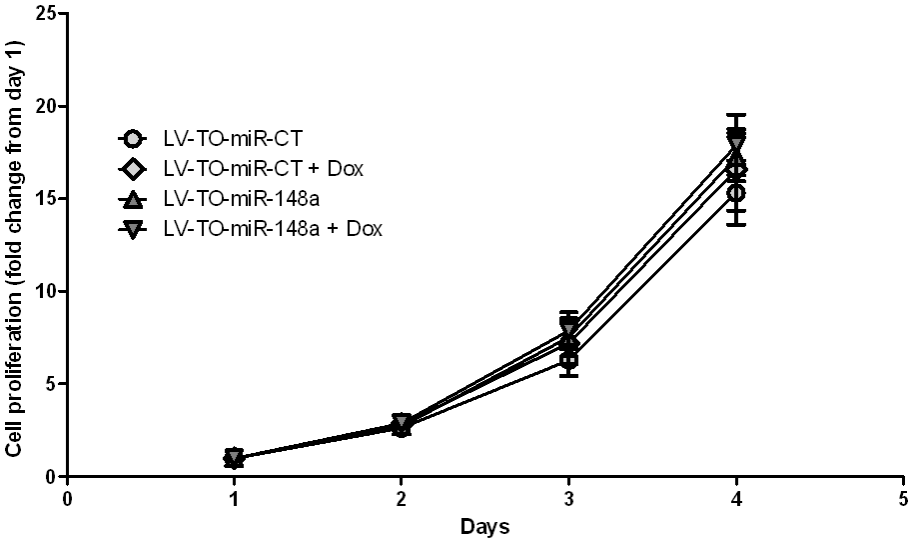
Effect of stable miR-148a over-expression in PDAC cell line. MIA PaCa-2 cells were incubated with inducible lentiviral vectors encoding for scramble microRNA (miR-CT) or miR-148a (miR-148a). MIA PaCa-2 miR-CT or miR-148a proliferation rate was assessed in the presence or the absence of doxycycline in culture medium. For each condition, cell count was performed every 24 h and compared to the number of adherent cells at day 1. The results are the mean of three independent experiments (±SEM).

In addition to transient transfection experiments, we generated Tet-ON-based lentiviral particles for stable expression of miR-148a to prevent rapid oligonucleotide decay and to allow a long term follow-up of cellular events in PDAC cell lines. We transducted MIA PaCa-2 cells due to their low expression level of endogenous miR-148a [7]. In these cells, doxycycline treatment leads to a five-fold increase in miR-148a expression, as compared to miR-CT over-expressing cells (Figure S1B). However, stable expression of miR-148a does not lead to significant changes in cellular proliferation in these cells, when compared to control cells (Figure 2). Additionally, migration and invasion potential of cells over-expressing miR-148a was measured and compared to control cells (Figure S2). Here, we observed that miR-148a over-expression does not modify these two cellular functions in our MIA PaCA-2 cell line model. Taken together, we demonstrate that similarly to transient over-expression, long-lasting expressions of miR-148a is ineffective for the inhibition of PDAC cell proliferation and does not impact cell behavior *in vitro*.

### Gemcitabine sensitivity of PDAC cell lines over-expressing miR-148a

**Figure 3:**
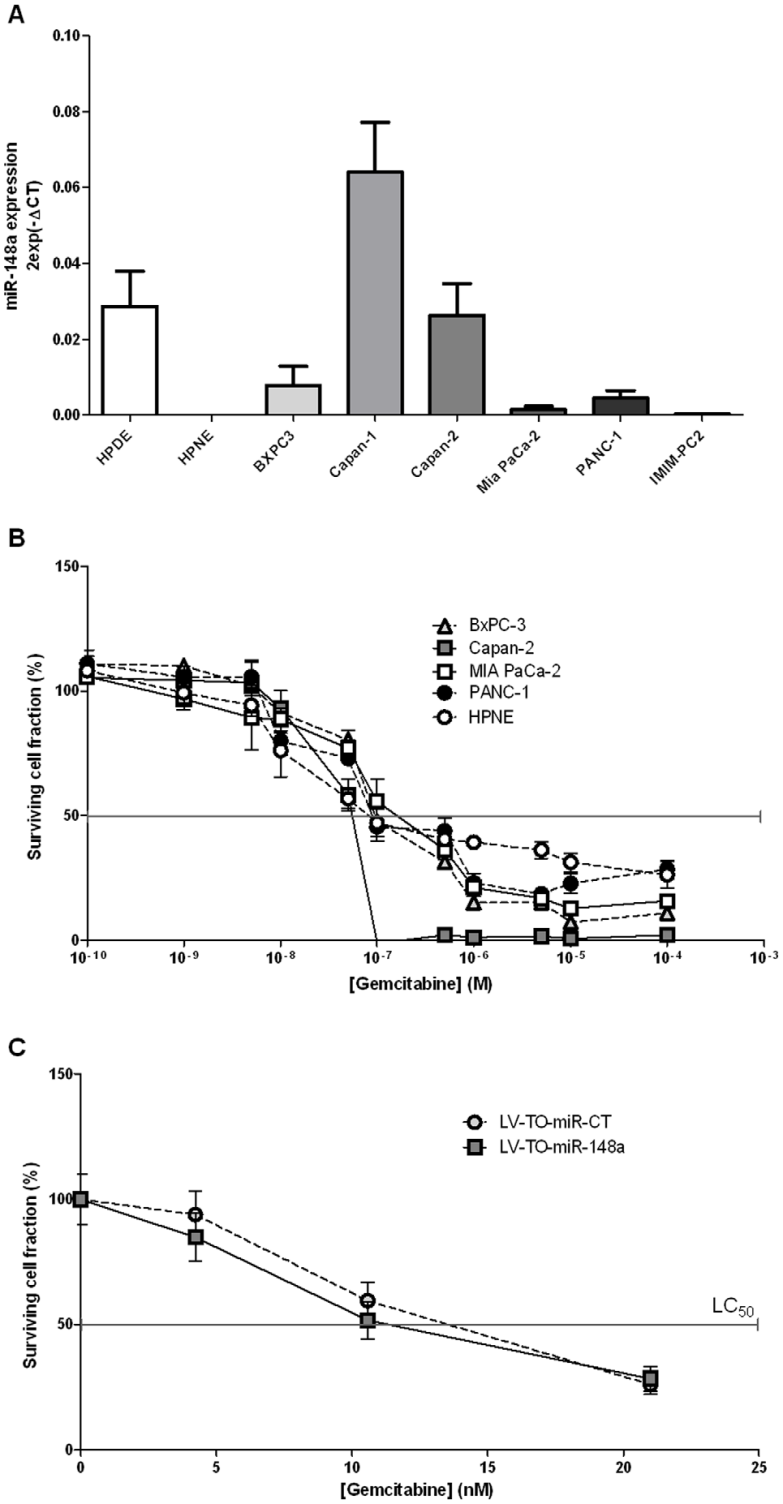
Gemcitabine sensitivity of PDAC cell lines over-expressing miR-148a. (**A**) miR-148a endogenous expression level was measured by qRT-PCR in several PDAC cell lines and in hPDE and hPNE normal pancreatic cell lines. (**B**) Cell sensitivity to gemcitabine was measured after a 72 h-treatment in several PDAC cell lines and in normal hPNE pancreatic cells. Surviving cell fraction represents the number of treated cells during 72 h compared to the number of untreated cells (represented as 100%). The lethal concentration 50 (LC50) represents the dose of gemcitabine sufficient to obtain 50% of surviving cells compared to untreated cells. The results are the mean of three independent experiments (±SEM). (**C**) Cell sensitivity to gemcitabine was measured in MIA PaCa-2 cells stably over-expressing miR-148a (LV-TO-miR-148a) or a control miR (LV-TO-miR-CT) to gemcitabine in presence of doxycycline after a 72 h-treatment. Surviving cell fraction represents the number of treated cells during 72 h compared to the number of untreated cells (represented as 100%). The results are the mean of three independent experiments (±SEM).

It has been proposed previously that microRNAs can affect chemosensitivity by targeting drug transporters or impairing cellular pathway crucial for drug metabolism or cell survival [16]. As described above, gemcitabine is the reference chemotherapy for PDAC. We assessed whether miR-148a participates to PDAC cells sensitivity against this drug. We first noticed no significant correlation between miR-148a endogenous level (Figure 3A) and gemcitabine sensitivity in several PDAC cell lines (Figure 3B). In parallel, we evaluated gemcitabine sensitivity in MIA PaCa-2 cells stably over-expressing miR-148a or a miR-CT (Figure 3C). Results obtained from three independent assays indicate a non significant decrease in the lethal concentration 50 value (LC50) of gemcitabine in the presence of miR-148a as compared to control cells. Similar results were obtained after transient transfection of miR-148a in several PDAC cell lines (Figure S3). Altogether, these results indicate that miR-148a does not dramatically influence PDAC cell lines sensitivity against gemcitabine.

### Protein expression profile analysis after transient miR-148a over-expression

Each microRNA can potentially affect the translation of dozens of mRNA targets according to the conservation of its target sequence [6]. The absence of cellular effects after miR-148a over-expression could be explained both by a weak change in protein expression profile, insufficient to produce a cellular effect, or by the up-regulation of a rescuing molecular pathway, consequent to miR-148a target decay. Consequently, we performed a large scale analysis of protein expression profiles by 2D-DIGE after transient miR-148a over-expression in Capan-2 cells. Capan-2 cells transfected with miR-CT were used as control. Fluorescence analysis of 2D-gels revealed that miR-148a over-expression only results in a faint modulation of approximately 2200 protein expression when compared to control cells (Figure S4). None of these variations reached the cut-off value (1.5 fold change as compared to control transfected cells) despite a massive over-expression of miR-148a (Figure S1A). In consequence, no protein species were identified by mass spectrometry.

These results indicate that miR-148a does not dramatically affect protein expression profiles in Capan-2 cells that may account for the absence of biological effects consequent to its over-expression.

### *In vivo* effects of miR-148a over-expression

To determine whether miR-148a may influence tumor growth *in vivo*, we generated orthotopic PDAC tumors in SCID CB17 mice using MIA PaCa-2 cells stably over-expressing miR-148a by 5 fold (Figure S1B). These cells constitutively express Gaussia luciferase (Gluc) for non-invasive tracking of tumor growth. As described by Chung and coworkers, Gluc quantification in serum accurately reflects the amount of viable cancer cells in xenograft tumors [17]. For *in vivo* studies, Gluc was sampled every 5 days and mice were sacrificed after 33 days, according to tumor size estimated by abdominal palpation. The correlation between tumor weight and Gluc signal (R^2^ = 0.79) was confirmed after tumor resection and comparison to Gluc dosage in serum sampled the day of surgery (Figure S5).

**Figure 4:**
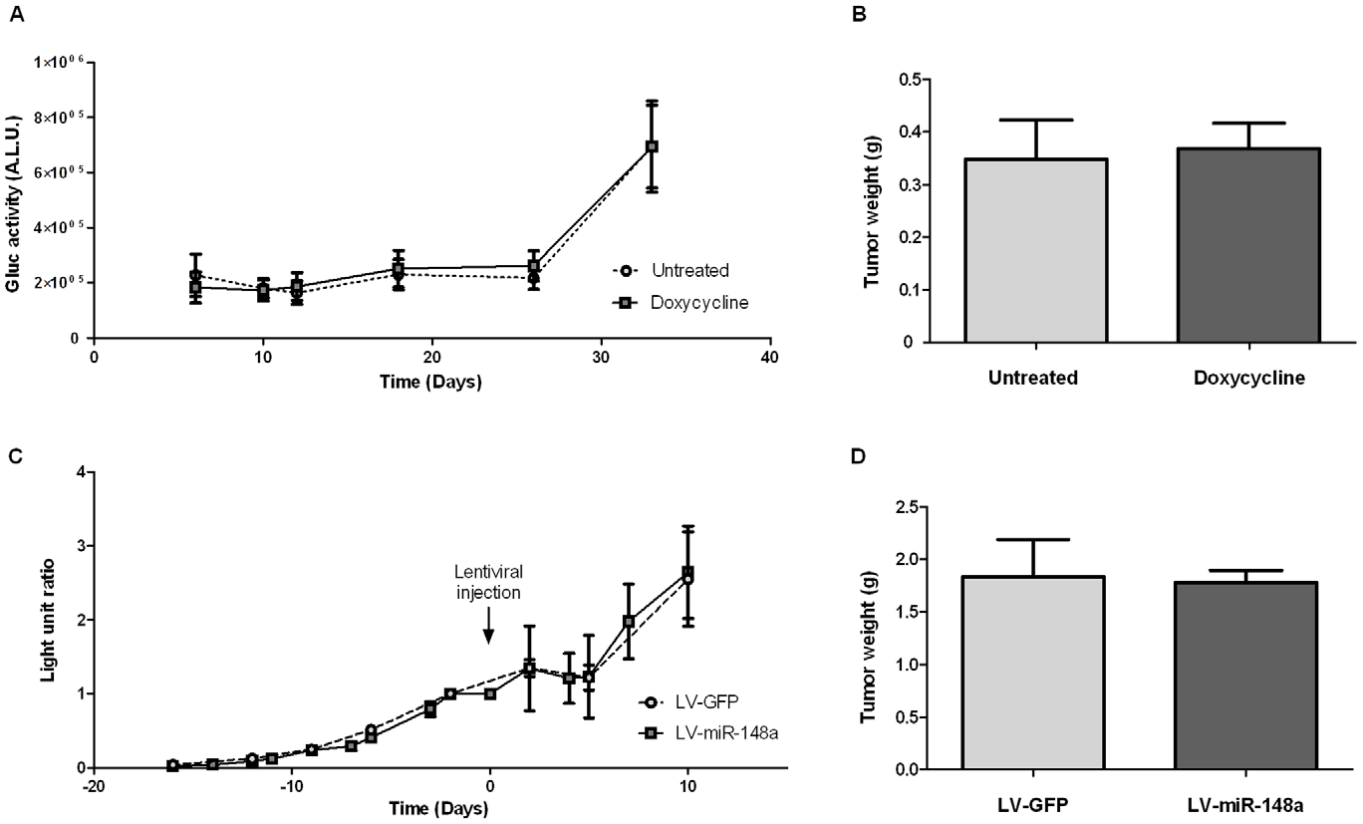
Murine orthotopic xenograft and tumor progression monitoring. (**A**) *In vivo* monitoring of xenograft tumor progression: MIA PaCa-2 cells expressing miR-148a and secreted Gaussia luciferase (Gluc) were injected in the pancreas of SCID mice. Mice received normal water (untreated, n = 10) or water supplemented with doxycycline (doxycycline, n = 12) for miR-148a expression induction until sacrifice. Gluc levels were measured in mice serum. The results are the mean of Gluc activities (±SEM) and are expressed as Arbitrary Light Unit (A.L.U.). (**B**) Tumors were removed and weighted the day of surgery. Results are mean (±SEM) of tumor weight in the untreated group (n = 10) and the doxycycline treated group (n = 12) (**C**) *In vivo* monitoring of xenograft tumor progression: MIA PaCa-2 cells expressing secreted Gaussia luciferase were injected in the pancreas of SCID mice (n = 10). Fifteen days later, lentiviral vectors encoding miR-148a (miR-148a, n = 7) or GFP only (GFP, n = 3) were injected in the tumors (the arrow indicates the lentiviral particles injection). The amount of Gluc was measured in mice serum and compared to the Gluc amount measured the day of lentivector injection (Day 0). The results are the mean of the Gluc level ratio in each group (±SEM) and are expressed as arbitrary light unit ratio. (**D**) Tumors receiving miR-148 (miR-148a, n = 7) or GFP (GFP, n = 3) were removed 10 days after lentiviral injection and were weighted. The graph represents the comparison of tumor weight expressing miR-148a (n = 7) or GFP (n = 3). The results are the mean of tumor weight in each group (±SEM).

During the course of this experiment, we found that Gluc levels were not altered by miR-148a expression indicating that miR-148a does not inhibit PDAC tumor progression *in vivo* (Figure 4A). Consistent with these findings, the analysis of tumors following surgery failed to reveal miR-148a inhibitory effects on tumor weight (Dox: 0.368 g±0.16 g *vs* Untreated: 0.348 g±0.22 g) (Figure 4B). Furthermore, histological study of the tumors indicates no difference in tissue organization between the different groups (Figure S6). These observations reveal no dramatic effect of miR-148a expression on tumor growth or tumor tissue development *in vivo*.

### *In vivo* measurement of miR-148a therapeutic potential

To assess an anti-tumor potential of an acute miR-148a over-expression in both tumor cells and the microenvironment, we injected lentiviral particles encoding miR-148a (LV-miR-148a) in pre-established MIA PaCa-2-Gluc tumors engrafted in the pancreas of SCID CB17 mice. Lentiviral particles encoding GFP reporter protein (LV-GFP) were used as control. MiR-148a over-expression in LV-miR-148a-injected tumors was verified by qRT-PCR (Figure S7). Tumor progression was monitored by Gluc sampling in mice serum. A decrease in Gluc signal was observed within 5 days after tumor transduction for both particles (Figure 4C). From day 5 to day 10, Gluc levels were strictly similar in miR-148a and control-transducted tumors, which indicate no difference in tumor viability following lentiviral vectors injection. In accordance with Gluc dosage, resected tumor weights at day 10 are similar in LV-miR-148a and LV-GFP-transducted groups (LV-miR-148a: 1.77 g±0.30 g *vs* LV-GFP: 1.97 g±0.63 g) (Figure 4D). These results indicate that miR-148a gene transfer in both tumor tissue and microenvironment does not impact tumor viability and reveals no particular therapeutic potential.

## Discussion

MiR-148a expression is altered in several types of cancer and its down-regulation is well described in various solid tumors such as colorectal, ovarian, prostate and gastric cancers [8], [11], [18], [19]. Our previous study reports that miR-148a expression is down-regulated by DNA hypermethylation of its genomic sequence [7]. This down-regulation occurs early in PDAC development and suggests a tumor suppressive effect. However, the role of miR-148a in pancreatic carcinogenesis has not been largely investigated yet. Liffers *et al.* are the only group that investigated the functions of miR-148a in PDAC cell lines [20]. Their work highlighted the targeting of cell-division-cycle-25-homolog-b (CDC25b) in several PDAC cells, raising the evidence of a miR-148a dependent control of cell cycle through G_2_/M checkpoint. Based on these results, our study was aimed at determining whether restoring miR-148a expression in PDAC cells could inhibit cell proliferation and tumor progression and therefore could be used as a therapeutic tool against PDAC.

We used both transient and stable miR-148a over-expression approaches in several PDAC cell lines. Despite significant miR-148a over-expression, we could not observe any growth inhibition of all PDAC cell lines tested *in vitro* and *in vivo*. This absence of effects contradicts Liffers' study on the potential of miR-148a to inhibit PDAC cell growth. However, this discrepancy can be explained by the fact that the miR-148a inhibitory effect described by Liffers *et al.* is only observed *in vitro* after a 100 fold-stable over-expression of miR-148a in IMIM-PC2 cells, which are rarely used as cellular model in PDAC studies and certainly display different genetic and epigenetic alterations than the PDAC cells used in our study.

At first sight, this absence of miR-148a effects on cell growth in a PDAC context also contradicts most of published articles on the role of miR-148a in other cancer. Indeed, Lujambio *et al.* demonstrated in SIHN-011B cell line that miR-148a over-expression suppresses tumor invasion and dissemination *in vitro* and *in vivo* [11]. In colorectal cancer cells, Zhang *et al.* showed a pro-apoptotic effect of miR-148a transient expression by the targeting of BCL-2 (B-cell lymphoma 2) mRNA [10]. Additionally, Fujita *et al.* described that transfection with miR-148a precursor inhibits cell growth, migration and invasion of prostate cancer-derived cells [19]. However, if we carefully read the literature, it is clear that miR-148a effects are cancer type-dependent or even more team-dependent. Indeed, a recent study revealed that the inhibition of miR-148a in HepG2 and Hep3B cells suppresses cell proliferation, cell cycle progression, cell migration *in vitro* and *in vivo* suggesting proliferative functions for miR-148a in hepatocellular carcinoma context [21]. Moreover, miR-148a down-regulation is described in gastric cancer by several groups [22], [23]. Interestingly, the consequence of miR-148a over-expression in gastric cancer-derived cell lines is variable among teams. Indeed, Zheng *et al.* demonstrated that the stable over-expression of miR-148a in human gastric cancer AGS and MGC-803 cell lines has no obvious effects on cell proliferation but suppresses tumor cell invasion and metastasis [8]. They therefore proposed a tumor suppressive function for miR-148a. Conversely, Guo's group established that miR-148a over-expression in the same AGS cell line leads to an increase in cell migration and proliferation suggesting an oncogenic effect [24]. Taken together, these data from the literature strongly force us to be extremely careful regarding the effects (or absence of effects) of microRNAs that greatly depend on cellular and experimental contexts.

MicroRNA are thought to impact chemo-sensitivity through the targeting of drug transporters or enzymes necessary for their metabolism [16]. MiR-148a was shown to influence cell chemo-sensitivity to paclitaxel, cisplatin and 5-FU in various cancer derived-cell lines although the molecular mechanisms remain unclear [9], [19]. Since gemcitabine is the reference chemotherapy against PDAC we assessed whether miR-148a participates to PDAC cell sensitivity against this drug. We first observed that no correlation exists between miR-148a endogenous expression level in several PDAC cell lines and intrinsic sensitivity to gemcitabine. Additionally, we showed that neither transient nor stable over-expression of miR-148a improves PDAC-derived cell lines sensitivity to gemcitabine *in vitro.*

The absence of miR-148a effects on cell growth and chemo-sensitivity in our PDAC cellular models prompted us to verify the functionality of our miR-148a expressing tools. At that time, several mRNA targets of miR-148a such as DNA methyltransferase 3 beta (DNMT3B), DNA methyltransferase 1 (DNMT1), BCL2 were described in various cancers using transient transfection and Western blot analysis [10], [25], [26]. Nevertheless, CDC25b was the only miR-148a target described in a PDAC-derived cell line [20]. Using qRT-PCR, luciferase reporter construct containing CDC25b 3′-UTR and Western blot, we ensured the proper expression and functionality of our mature miR-148a expressing tools (Figures S1C and S1D), which are in accordance with Liffers' observations. These results demonstrate that the absence of miR-148a effects on cell growth and chemoresistance was not due to an impairment of miR-148a constructs used in the present study.

We next decided to perform a large scale proteomic analysis. Indeed, the targeting of several molecular pathways could lead to a cross inhibition and explain the marginal effect of this microRNA in our PDAC cellular context. To our knowledge, only two groups have performed 2D-DIGE analysis after transfection of microRNA precursors *in vitro* [27], [28]. Our results are consistent with these studies which indicate that microRNAs are fine tuning molecules which modestly modulate protein expression. Our 2D-DIGE approach did not reveal changes in protein expression that reach the common 1.5 fold cut-off value suggesting that our miR-148a over-expression in PDAC cellular context faintly modifies protein expression profiles. This is in accordance with the 30% decrease of CDC25b protein expression in response to miR-148a transient over-expression (Figure S1D).

Altogether our *in vitro* results strongly suggest that delivering either plasmid-driven gene or synthetic miR-148a oligonucleotides is not sufficient to counteract the cancerous phenotype of four well described PDAC-derived cell lines in contrary to other cancer-type derived cell lines. This can be explained by the extreme aggressiveness of PDAC-derived cell lines that have developed a molecular arsenal to resist to all tested chemotherapy.

We finally asked whether miR-148a could exert a tumor suppressive effect in a preclinical PDAC *in vivo* model. Xenograft of miR-148a over-expressing cells has been already performed in colorectal, gastric and head and neck cancer [8], [10], [11]. Consequences were highly variables as they promoted or decreased tumor progression depending on cancer type. Here, we used similar strategies of either stable or acute *in vivo* miRNA over-expression that we previously described for *let-7* in PDAC tumors [15]. The first approach of xenograft we describe herein consisted in the injection of MIA PaCa-2 cells overexpressing miR-148a under doxycycline treatment. In accordance with our *in vitro* findings demonstrating that miR-148a has no clear effect on cell proliferation or survival of PDAC cells, we describe that miR-148a does not impede tumor engraftment and *in vivo* progression.

The second *in viv*o approach consisted in the transduction of pre-established Mia PaCa-2 tumors, with miR-148a expressing lentiviral particles. This strategy permits an acute miR-148a expression in both tumor cells and tumor microenvironment. Despite a weak decrease in tumor progression in both groups 5 days following transduction, over-expressing miR-148a in PDAC did not inhibit tumor progression. Determination of miR-148a over-expression *in vivo* is challenging, considering the strict conservation of both miR-148a precursor and mature sequence between mouse and human genome and the considerable part of murine cells participating to tumor formation in orthotopic xenograft model. Despite these experimental barriers, the injection of viral particles in Mia PaCa-2 tumors leads to a clear increase in miR-148a expression in the tumors (Figure S7).

Taken together, our *in vivo* experiments show that miR-148a over-expression fails to inhibit PDAC tumor engraftment and *in vivo* progression. Consequently, we conclude that miR-148a restoration is not an appropriate therapeutic target for the management of advanced PDAC.

## Supporting Information

Figure S1 **Functionality of miR-148a over-expressing tools.** MiR-148a expression level was measured by qRT-PCR following: (**A**) transient transfection in Capan-2 cells using a miR-148a precursor oligonucleotide or a control oligonucleotide, (**B**) stable transduction of MIA PaCA-2 cells using LV-TO-miR-148a or LV-TO-CT or (**C**) stable transduction of MIA PaCA-2 cells using LV-miR-148a or LV–GFP. (**D**) To determine whether the miR-148a encoded by our different tools is properly processed and functional, the targeting of CDC25b 3′-UTR is measured using a luciferase reporter construct as described by Liffers *et al.*. Results are expressed as percentage of luciferase signal compared to control cells. (**E**) The expression of cdc25b protein was assessed by Western blot after transient over-expression of miR-148a in IMIM-PC2 PDAC cells as described by Liffers *et al.* The graph represents the quantification of three independent Western blot experiments. Results are expressed as percentage of CDC25b protein expression compared to miR-CT transfected cells.
(PDF)

Figure S2 **Migration (A) and invasion (B) capacity of miR-148a over-expressing cells.** One hundred thousand exponentially growing cells over-expressing miR-148a or GFP were starved for 24 h and seeded into 8 µm trans-wells non-coated (migration test) or coated (invasion test) with matrigel. After 15 h, migrated cells were stained; lysed and cellular density was determined by optical density measure of cell lysates at 560 nm. Graphs represent results of three independent experiments and are expressed as percentage of migrating or invading miR-148a over-expressing cells compared to GFP expressing cells.
(PDF)

Figure S3 **Gemcitabine sensitivity of PDAC cell lines.** One thousand of exponentially growing PDAC cells transiently over-expressing miR-148a or control microRNA (miR-CT) were plated in 96 well plate. Cells were treated with different doses of gemcitabine ranging from 10^−9^ M to 10^−4^ M for 72 h. For each cell line, cell viability was assessed by colorimetric method, compared to viability measured in 10^−9^ M treated wells and represented as percentage of surviving cells.
(PDF)

Figure S4 **Two Dimensions-Gel Electrophoresis proteomic analysis after transient miR-148a over-expression in Capan-2 cells.** Capan-2 cells were transiently transfected with miR-CT or miR-148a as described in Materials and Methods section (n = 4). Proteins were extracted, differentially labeled with cyanines and ran depending on their charge and their isoelectric point as described in Materials and Methods section. For each gel, spot variation between our two conditions was measured using DeCyder 6.5 software (GE Healthcare).
(PDF)

Figure S5 **Correlation analysis between Gluc serum content and tumor weight.** Orthotopic MIA PaCa-2-Gluc cell xenografts were performed in SCID mice as described in Materials and Methods section (n = 24). Thirty three days after injection, tumors were removed; scaled and 100 µl of blood was sampled with 10 µl of a 20% EDTA solution. Serum was isolated and Gluc content was assessed as described in Materials and Methods section. A regressive linear correlation was calculated to assess the correspondence between Gluc serum level and tumor weight and displayed a R^2^ = 0.79.
(PDF)

Figure S6 **Histological organization of grafted tumors.** Twelve million of exponentially growing MIA PaCa-2 cells stably over-expressing miR-148a were injected in the tail of the pancreas of SCID mice. Mice grafted with miR-148a expressing cells (doxycycline) received water *ad libitum* supplemented with sucrose (25 g/L) and doxycycline (2 g/L). Control mice (untreated) received water *ad libitum* supplemented with sucrose only (25 g/L). Thirty days after xenograft, mice were sacrificed and tumors were removed, weighed and measured. Anatomopathological examination of tumors revealed no difference in histological organization, characterized by a classic fibrous capsule (**A**) surrounding a necrotic core (**B**), lined by a thin layer of tumor cells (**C**).
(PDF)

Figure S7 **MiR-148a expression after i** ***n vivo*** **tumor transduction of MIA PaCa-2 tumors.** Orthotopic tumors were established in SCID CB17 mice pancreas by injection of 12×10^6^ of exponentially growing MIA PaCa-2-Gluc and were injected with lentiviral particles encoding miR-148a or the GFP reporter protein (as described in Materials and Methods section). After removal, whole tumors were homogenized in TRIzol Reagent. Total RNAs were extracted and miR-148a expression was determined by qRT-PCR using specific primers directed against mature miR-148a and U6 RNA used for normalization. Relative amounts of miRNA were calculated by the comparative threshold cycle (CT) method.
(PDF)
