# Supplementary material for: The Rescue of miR-148a Expression in Pancreatic Cancer: An Inappropriate Therapeutic Tool
Source: PLoS One. 2013 Jan 31;8(1):e55513. doi: 10.1371/journal.pone.0055513 (PMC3561221; doi:10.1371/journal.pone.0055513)
Supplement: Figure S1 — Functionality of miR-148a over-expressing tools. MiR-148a expression level was measured by qRT-PCR following: (A) transient transfection in Capan-2 cells using a miR-148a precursor oligonucleotide or a control oligonucleotide, (B) stable transduction of MIA PaCA-2 cells using LV-TO-miR-148a or LV-TO-CT or (C) stable transduction of MIA PaCA-2 cells using LV-miR-148a or LV–GFP. (D) To determine whether the miR-148a encoded by our different tools is properly processed and functional, the targeting of CDC25b 3′-UTR is measured using a luciferase reporter construct as described by Liffers et al.. Results are expressed as percentage of luciferase signal compared to control cells. (E) The expression of cdc25b protein was assessed by Western blot after transient over-expression of miR-148a in IMIM-PC2 PDAC cells as described by Liffers et al. The graph represents the quantification of three independent Western blot experiments. Results are expressed as percentage of CDC25b protein expression compared to miR-CT transfected cells. (PDF) [file pone.0055513.s001.pdf]

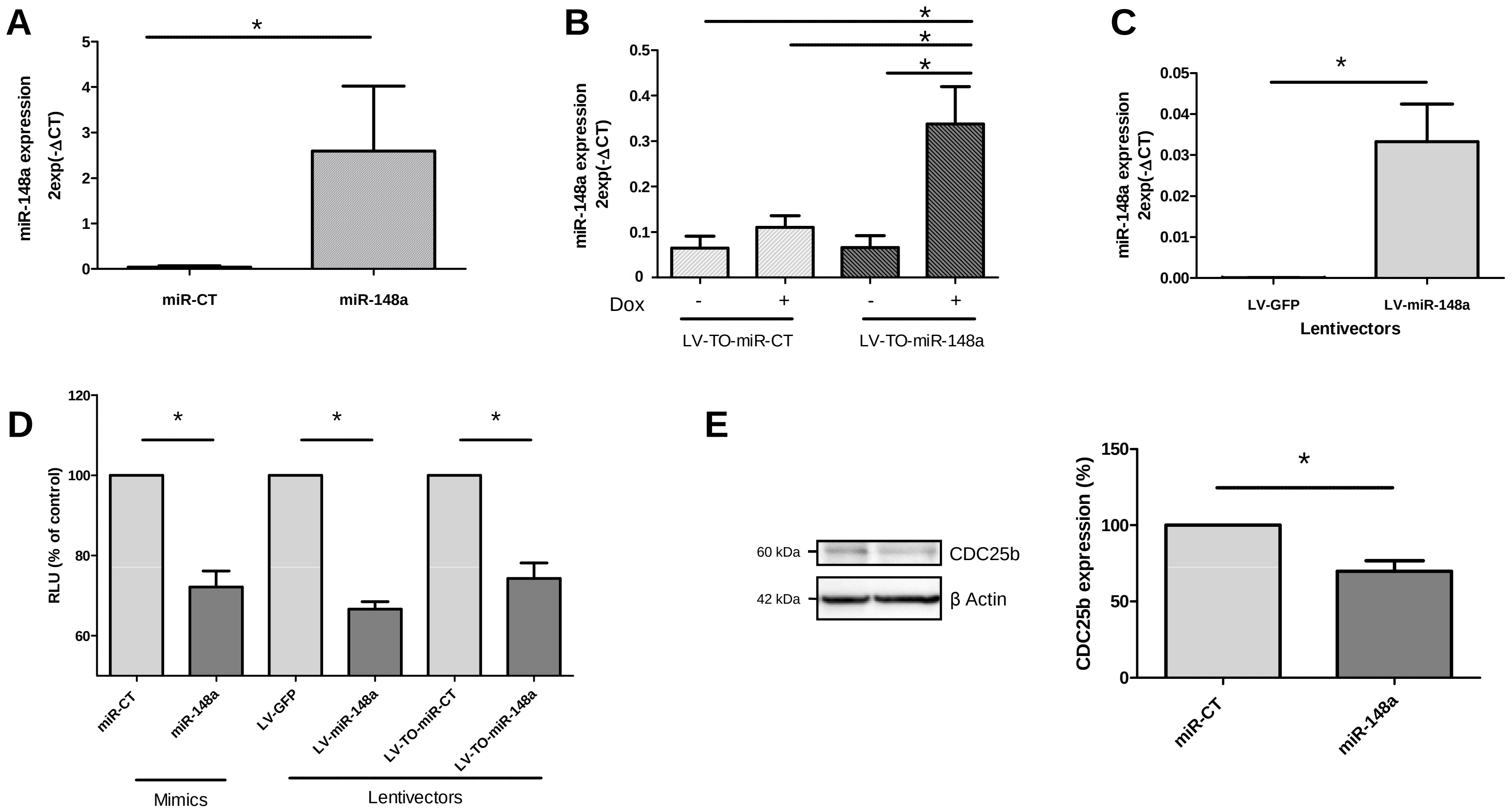

Supplementary Figure 1. Functionality of miR-148a over-expressing tools. MiR-148a expression level was measured by qRT-PCR following : **(A)** transient transfection in Capan-2 cells using a miR-148a precursor oligonucleotide or a control oligonucleotide, **(B)** stable transduction of MIA PaCA-2 cells using LV-TO-miR-148a or LV-TO-CT or **(C)** stable transduction of MIA PaCA-2 cells using LV-miR-148a or LV–GFP. **(D)** To determine whether the miR-148a encoded by our different tools is properly processed and functional, the targeting of CDC25b 3'-UTR is measured using a luciferase reporter construct as described by Liffers *et al.*. Results are expressed as percentage of luciferase signal compared to control cells. **(E)** The expression of cdc25b protein was assessed by Western blot after transient over-expression of miR-148a in IMIM-PC2 PDAC cells as described by Liffers *et al.*. The graph represents the quantification of three independent Western blot experiments. Results are expressed as percentage of CDC25b protein expression compared to miR-CT transfected cells.
