# Supplementary material for: The Rescue of miR-148a Expression in Pancreatic Cancer: An Inappropriate Therapeutic Tool
Source: PLoS One. 2013 Jan 31;8(1):e55513. doi: 10.1371/journal.pone.0055513 (PMC3561221; doi:10.1371/journal.pone.0055513)
Supplement: Figure S2 — Migration (A) and invasion (B) capacity of miR-148a over-expressing cells. One hundred thousand exponentially growing cells over-expressing miR-148a or GFP were starved for 24 h and seeded into 8 µm trans-wells non-coated (migration test) or coated (invasion test) with matrigel. After 15 h, migrated cells were stained; lysed and cellular density was determined by optical density measure of cell lysates at 560 nm. Graphs represent results of three independent experiments and are expressed as percentage of migrating or invading miR-148a over-expressing cells compared to GFP expressing cells. (PDF) [file pone.0055513.s002.pdf]

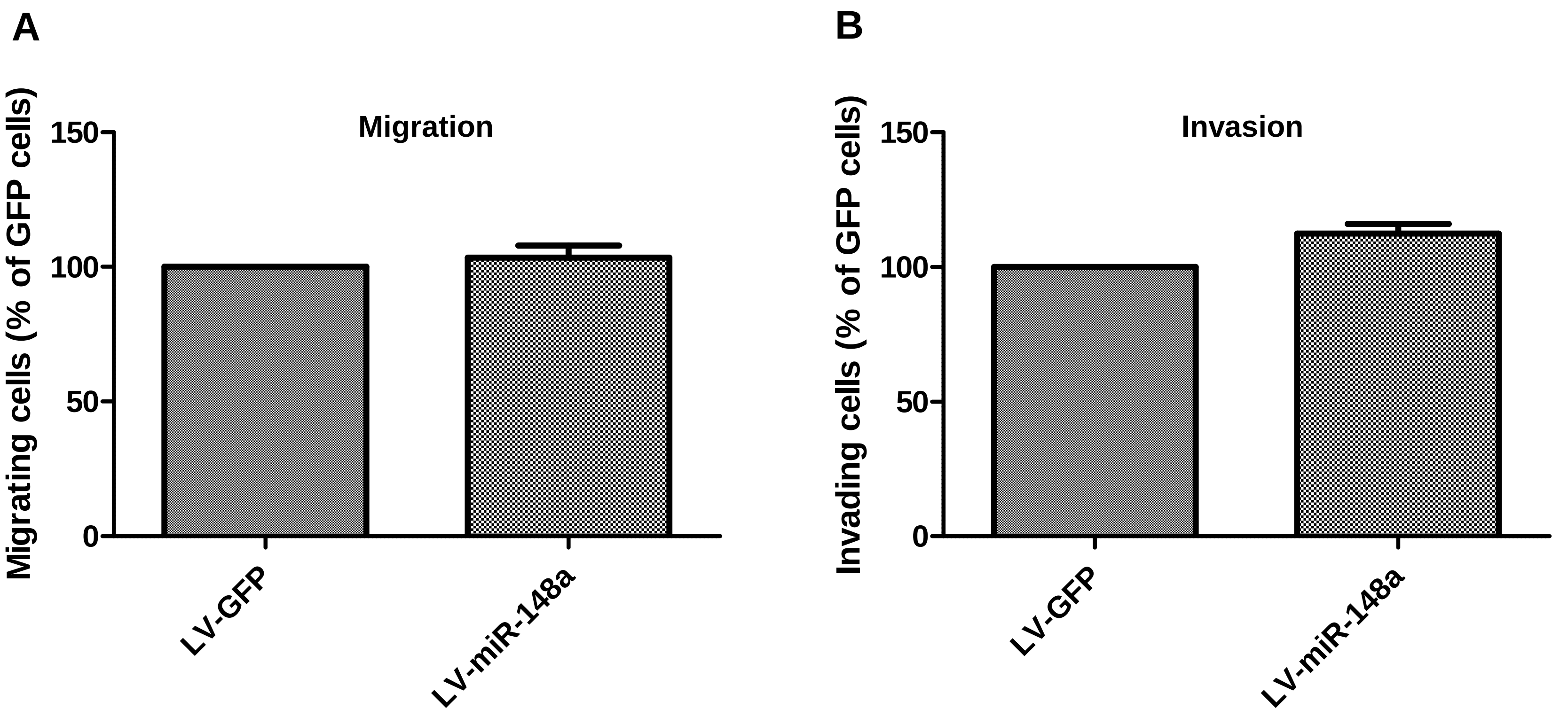

Supplementary Figure 2. Migration (A) and invasion (B) capacity of miR-148a over-expressing cells. One hundred thousand exponentially growing cells over-expressing miR-148a or GFP were starved for 24h and seeded into 8 µm transwells non-coated (migration test) or coated (invasion test) with matrigel. After 15h, migrated cells were stained, lysed and cellular density was determined by optical density measure of cell lysates at 560 nm. Graphs represent results of three independent experiments and are expressed as percentage of migrating or invading miR-148a over-expressing cells compared to GFP expressing cells.
