# Supplementary material for: The Rescue of miR-148a Expression in Pancreatic Cancer: An Inappropriate Therapeutic Tool
Source: PLoS One. 2013 Jan 31;8(1):e55513. doi: 10.1371/journal.pone.0055513 (PMC3561221; doi:10.1371/journal.pone.0055513)
Supplement: Figure S3 — Gemcitabine sensitivity of PDAC cell lines. One thousand of exponentially growing PDAC cells transiently over-expressing miR-148a or control microRNA (miR-CT) were plated in 96 well plate. Cells were treated with different doses of gemcitabine ranging from 10−9 M to 10−4 M for 72 h. For each cell line, cell viability was assessed by colorimetric method, compared to viability measured in 10−9 M treated wells and represented as percentage of surviving cells. (PDF) [file pone.0055513.s003.pdf]

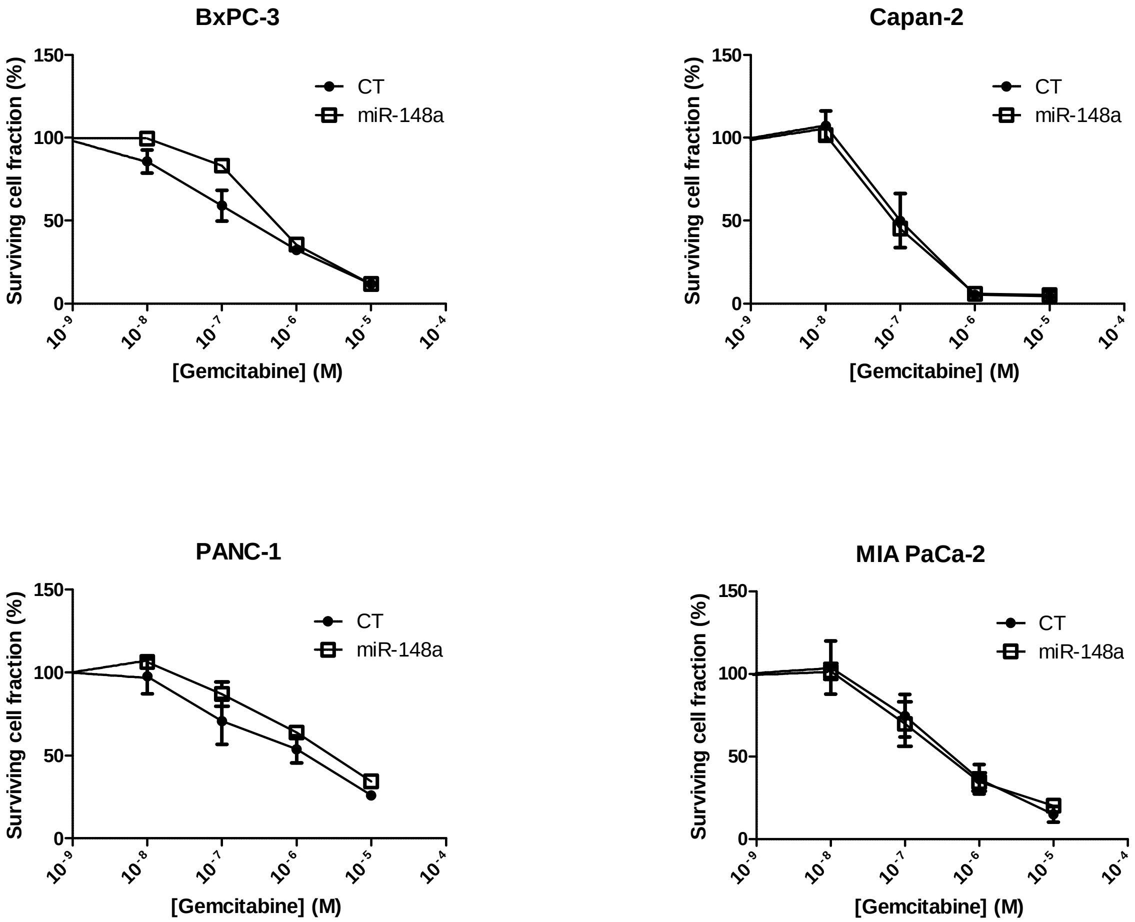

Supplementary Figure 3. Gemcitabine sensitivity of PDAC cell lines. One thousand of exponentially growing PDAC cells transiently over-expressing miR-148a or a control microRNA (miR-CT) were plated in 96 well plate. Cells were treated with different doses of gemcitabine ranging from 10^{-9} M to 10^{-4} M for 72h. For each cell line, cell viability was assessed by colorimetric method, compared to viability measured in 10^{-9}M treated wells and represented as percentage of surviving cells.
