# Supplementary material for: The Rescue of miR-148a Expression in Pancreatic Cancer: An Inappropriate Therapeutic Tool
Source: PLoS One. 2013 Jan 31;8(1):e55513. doi: 10.1371/journal.pone.0055513 (PMC3561221; doi:10.1371/journal.pone.0055513)
Supplement: Figure S4 — Two Dimensions-Gel Electrophoresis proteomic analysis after transient miR-148a over-expression in Capan-2 cells. Capan-2 cells were transiently transfected with miR-CT or miR-148a as described in Materials and Methods section (n = 4). Proteins were extracted, differentially labeled with cyanines and ran depending on their charge and their isoelectric point as described in Materials and Methods section. For each gel, spot variation between our two conditions was measured using DeCyder 6.5 software (GE Healthcare). (PDF) [file pone.0055513.s004.pdf]

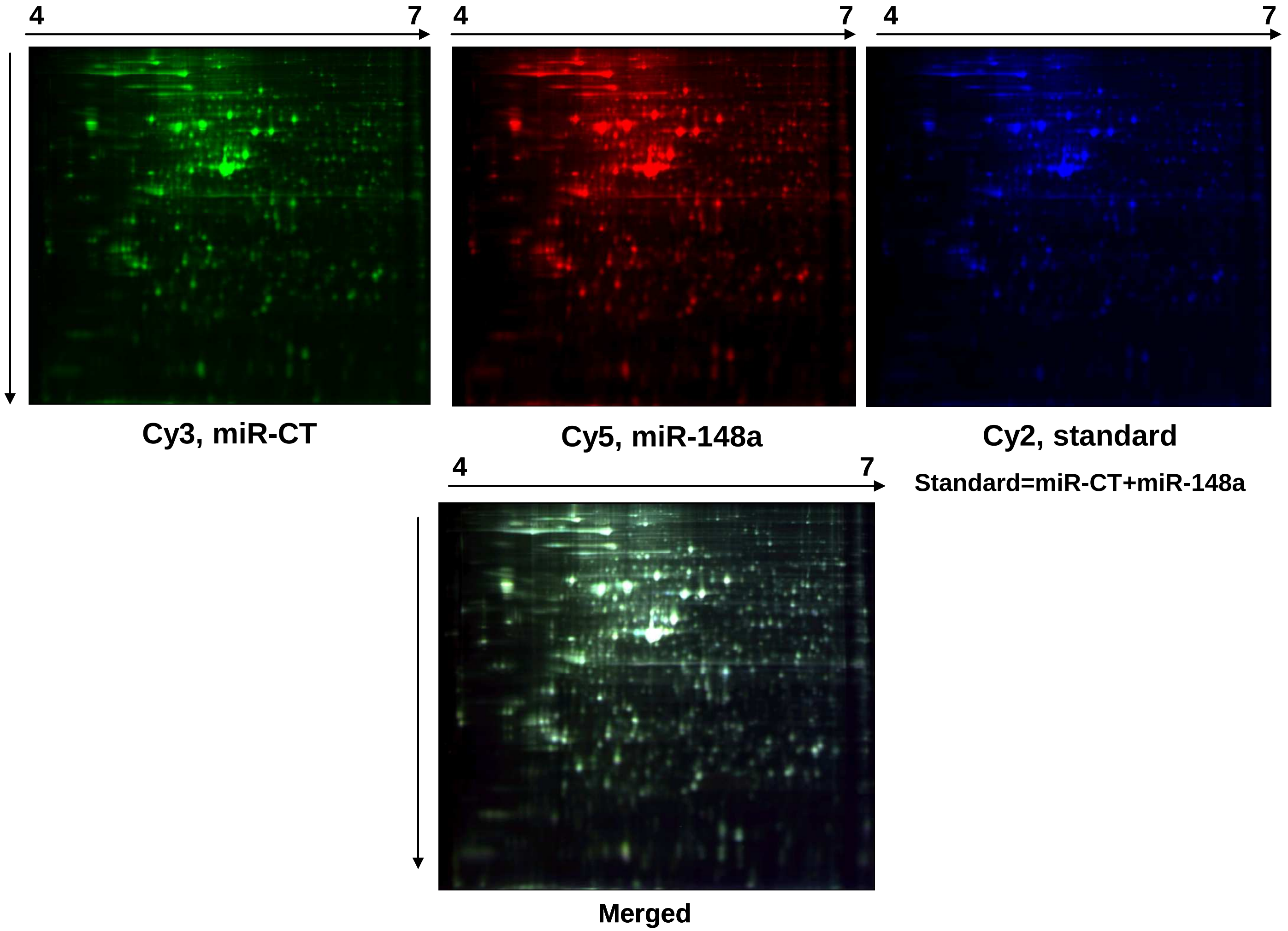

Supplementary Figure 4. Two Dimensions-Gel Electrophoresis proteomic analysis after transient miR-148a over-expression in Capan-2 cells. Capan-2 cells were transiently transfected with miR-CT or miR-148a as described in Materials and Methods section (n=4) . Proteins were extracted, differentially labeled with cyanines and ran depending on their charge and their isoelectric point as described in Materials and Methods section. For each gel, spot variation between our two conditions was measured using DeCyder 6.5 software (GE Healthcare).
