# Supplementary material for: The Rescue of miR-148a Expression in Pancreatic Cancer: An Inappropriate Therapeutic Tool
Source: PLoS One. 2013 Jan 31;8(1):e55513. doi: 10.1371/journal.pone.0055513 (PMC3561221; doi:10.1371/journal.pone.0055513)
Supplement: Figure S5 — Correlation analysis between Gluc serum content and tumor weight. Orthotopic MIA PaCa-2-Gluc cell xenografts were performed in SCID mice as described in Materials and Methods section (n = 24). Thirty three days after injection, tumors were removed; scaled and 100 µl of blood was sampled with 10 µl of a 20% EDTA solution. Serum was isolated and Gluc content was assessed as described in Materials and Methods section. A regressive linear correlation was calculated to assess the correspondence between Gluc serum level and tumor weight and displayed a R2 = 0.79. (PDF) [file pone.0055513.s005.pdf]

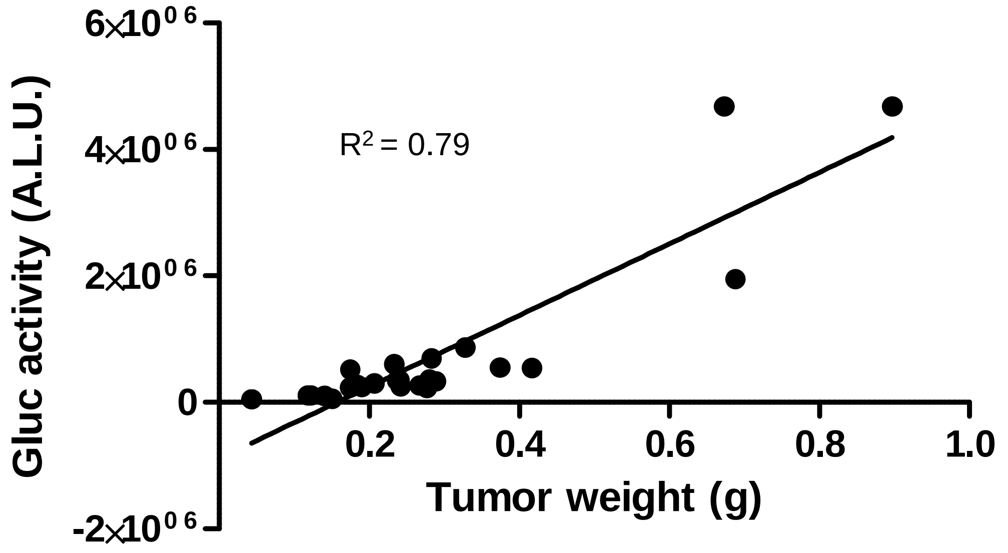

Supplementary Figure 5. Correlation analysis between Gluc serum content and tumor weight. Orthotopic MIA PaCa-2-Gluc cell xenografts were performed in SCID mice as described in Materials and Methods section (n=24). Thirty three days after injection, tumors were removed, scaled and 100 µl of blood was sampled with 10 µl of a 20% EDTA solution. Serum was isolated and Gluc content was assessed as described in Materials and Methods section. A regressive linear correlation was calculated to assess the correspondence between Gluc serum level and tumor weight and displayed a R^2=0.79.
