# Supplementary material for: The Rescue of miR-148a Expression in Pancreatic Cancer: An Inappropriate Therapeutic Tool
Source: PLoS One. 2013 Jan 31;8(1):e55513. doi: 10.1371/journal.pone.0055513 (PMC3561221; doi:10.1371/journal.pone.0055513)
Supplement: Figure S6 — Histological organization of grafted tumors. Twelve million of exponentially growing MIA PaCa-2 cells stably over-expressing miR-148a were injected in the tail of the pancreas of SCID mice. Mice grafted with miR-148a expressing cells (doxycycline) received water ad libitum supplemented with sucrose (25 g/L) and doxycycline (2 g/L). Control mice (untreated) received water ad libitum supplemented with sucrose only (25 g/L). Thirty days after xenograft, mice were sacrificed and tumors were removed, weighed and measured. Anatomopathological examination of tumors revealed no difference in histological organization, characterized by a classic fibrous capsule (A) surrounding a necrotic core (B), lined by a thin layer of tumor cells (C). (PDF) [file pone.0055513.s006.pdf]

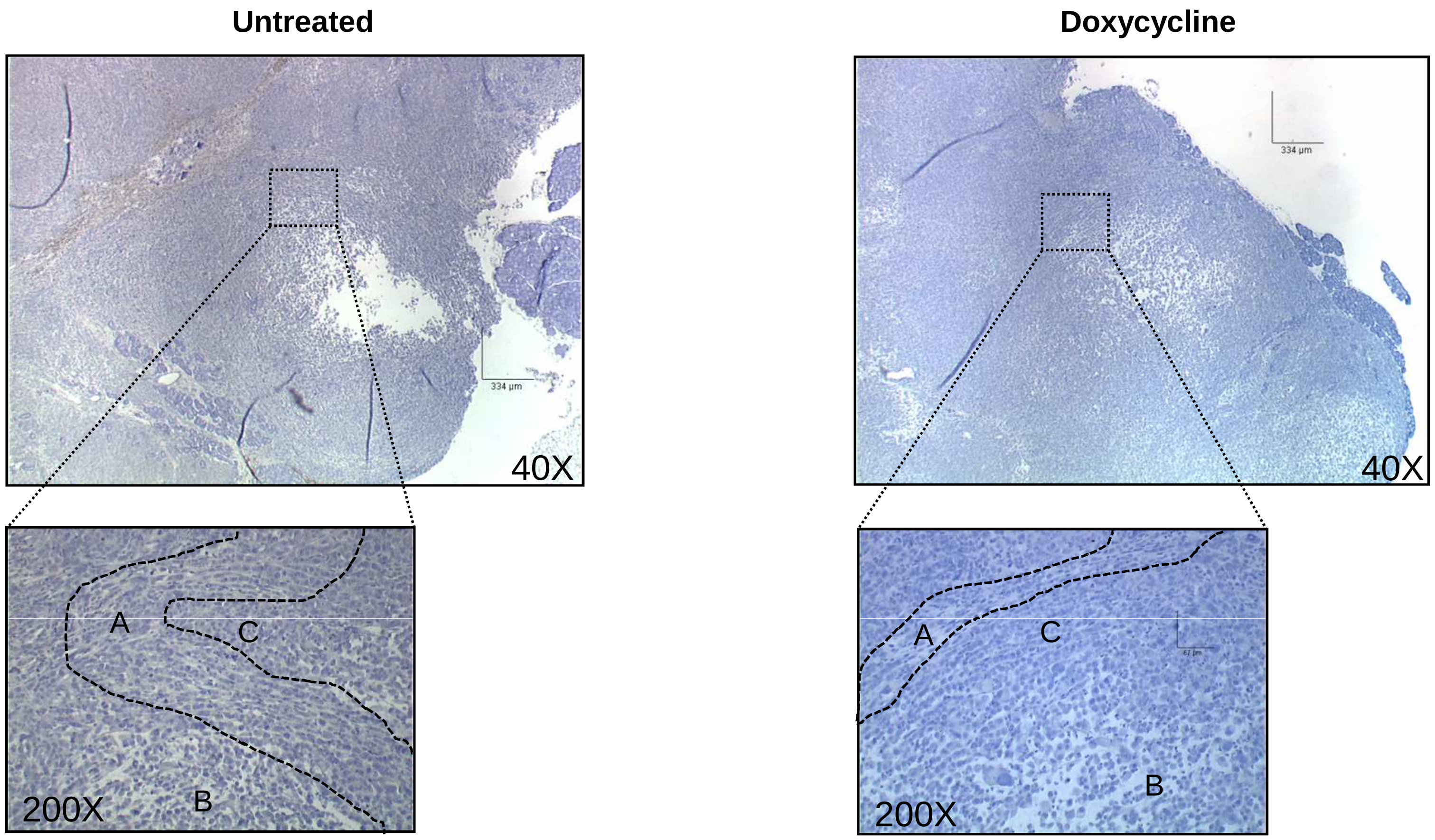

Supplementary Figure 6. Histological organization of grafted tumors. Twelve million of exponentially growing MIA PaCa-2 cells stably over-expressing miR-148a were injected in the tail of the pancreas of SCID mice. Mice grafted with miR-148a expressing cells (doxycycline) received water *ad libitum* supplemented with sucrose (25 g/L) and doxycycline (2 g/L). Control mice (untreated) received water *ad libitum* supplemented with sucrose only (25 g/L). Thirty days after xenograft, mice were sacrificed and tumors were removed, weighed and measured. Anatomopathological examination of tumors revealed no difference in histological organization, characterized by a classic fibrous capsule **(A)** surrounding a necrotic core **(B)**, lined by a thin layer of tumor cells **(C)**.
