# Supplementary material for: The Rescue of miR-148a Expression in Pancreatic Cancer: An Inappropriate Therapeutic Tool
Source: PLoS One. 2013 Jan 31;8(1):e55513. doi: 10.1371/journal.pone.0055513 (PMC3561221; doi:10.1371/journal.pone.0055513)
Supplement: Figure S7 — MiR-148a expression after i n vivo tumor transduction of MIA PaCa-2 tumors. Orthotopic tumors were established in SCID CB17 mice pancreas by injection of 12×106 of exponentially growing MIA PaCa-2-Gluc and were injected with lentiviral particles encoding miR-148a or the GFP reporter protein (as described in Materials and Methods section). After removal, whole tumors were homogenized in TRIzol Reagent. Total RNAs were extracted and miR-148a expression was determined by qRT-PCR using specific primers directed against mature miR-148a and U6 RNA used for normalization. Relative amounts of miRNA were calculated by the comparative threshold cycle (CT) method. (PDF) [file pone.0055513.s007.pdf]

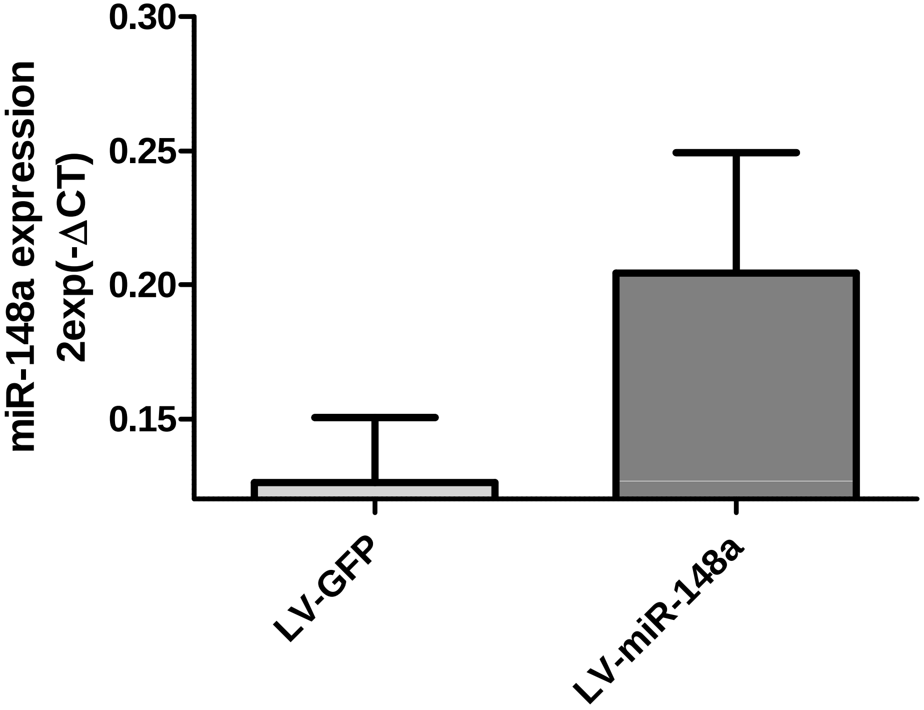

Supplementary Figure 7. MiR-148a expression after i*n vivo* tumor transduction of MIA PaCa-2 tumors. Orthotopic tumors were established in SCID CB17 mice pancreas by injection of 12x10^6 of exponentially growing MIA PaCa-2-Gluc and were injected with lentiviral particles encoding miR-148a or the GFP reporter protein (as described in Materials and Methods section). After removal, whole tumors were homogenized in TRIzol Reagent. Total RNAs were extracted and miR-148a expression was determined by qRT-PCR using specific primers directed against mature miR-148a and U6 RNA used for normalization. Relative amounts of miRNA were calculated by the comparative threshold cycle (CT) method.
